# Microglial extracellular vesicles induce Alzheimer’s disease-related cortico-hippocampal network dysfunction

**DOI:** 10.1093/braincomms/fcad170

**Published:** 2023-05-31

**Authors:** Chiara Falcicchia, Francesca Tozzi, Martina Gabrielli, Stefano Amoretti, Greta Masini, Gabriele Nardi, Stefano Guglielmo, Gian Michele Ratto, Ottavio Arancio, Claudia Verderio, Nicola Origlia

**Affiliations:** National Research Council (CNR) Institute of Neuroscience, Pisa 56124, Italy; National Research Council (CNR) Institute of Neuroscience, Pisa 56124, Italy; Bio@SNS laboratory, Scuola Normale Superiore, Pisa 56124, Italy; National Research Council (CNR) Institute of Neuroscience, Vedano al Lambro, Monza (MB) 20854, Italy; National Research Council (CNR) Institute of Neuroscience, Pisa 56124, Italy; National Research Council (CNR) Institute of Neuroscience, Pisa 56124, Italy; National Enterprise for nanoScience and nanoTechnology (NEST), Istituto Nanoscienze, Consiglio Nazionale delle Ricerche (CNR) and Scuola Normale Superiore Pisa, Pisa 56127, Italy; National Research Council (CNR) Institute of Neuroscience, Pisa 56124, Italy; Bio@SNS laboratory, Scuola Normale Superiore, Pisa 56124, Italy; National Enterprise for nanoScience and nanoTechnology (NEST), Istituto Nanoscienze, Consiglio Nazionale delle Ricerche (CNR) and Scuola Normale Superiore Pisa, Pisa 56127, Italy; Department of Pathology and Cell Biology, The Taub Institute for Research on Alzheimer’s Disease and the Aging Brain and Department of Medicine, Columbia University, New York, NY 10032, USA; National Research Council (CNR) Institute of Neuroscience, Vedano al Lambro, Monza (MB) 20854, Italy; National Research Council (CNR) Institute of Neuroscience, Pisa 56124, Italy

**Keywords:** extracellular vesicles, entorhinal cortex, Alzheimer’s disease, microglia, cortical-hippocampal network

## Abstract

β-Amyloid is one of the main pathological hallmarks of Alzheimer’s disease and plays a major role in synaptic dysfunction. It has been demonstrated that β-amyloid can elicit aberrant excitatory activity in cortical-hippocampal networks, which is associated with behavioural abnormalities. However, the mechanism of the spreading of β-amyloid action within a specific circuitry has not been elucidated yet. We have previously demonstrated that the motion of microglia-derived large extracellular vesicles carrying β-amyloid, at the neuronal surface, is crucial for the initiation and propagation of synaptic dysfunction along the entorhinal–hippocampal circuit. Here, using chronic EEG recordings, we show that a single injection of extracellular vesicles carrying β-amyloid into the mouse entorhinal cortex could trigger alterations in the cortical and hippocampal activity that are reminiscent of those found in Alzheimer’s disease mouse models and human patients. The development of EEG abnormalities was associated with progressive memory impairment as assessed by an associative (object-place context recognition) and non-associative (object recognition) task. Importantly, when the motility of extracellular vesicles, carrying β-amyloid, was inhibited, the effect on network stability and memory function was significantly reduced. Our model proposes a new biological mechanism based on the extracellular vesicles–mediated progression of β-amyloid pathology and offers the opportunity to test pharmacological treatments targeting the early stages of Alzheimer’s disease.

## Teaser

Falcicchia *et al*. report that when injected into the mouse entorhinal cortex, large extracellular vesicles that are isolated from microglia, previously exposed to β-amyloid, could trigger cortico-hippocampal network hyperexcitability. These EEG alterations are reminiscent of those found in Alzheimer’s disease models and human patients and were associated with progressive memory impairment.

## Graphical Abstract

**Graphical Abstract:**
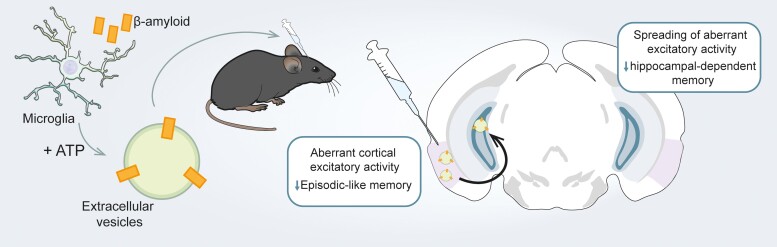

## Introduction

Pathological hallmarks of Alzheimer’s disease (AD) have been investigated in depth, but the relationship between β-amyloid and cognitive deficits is still poorly understood. According to the ‘amyloid hypothesis’, Aβ plays a major role in synaptic dysfunction, particularly in its oligomeric form.^1–3^ Palop and Mucke^2^ demonstrated, by EEG recordings, that the spreading of Aβ pathology within a specific circuitry (i.e. entorhinal cortex/hippocampal connections) triggers cortico-hippocampal hyperexcitability, establishing a link between Aβ propagation, hyperexcitability initiation and the development of behavioural abnormalities.^4^ Moreover, recent evidence suggests that the spreading of synaptic dysfunction and subsequent aberrant network activity can involve microglial cells.^5–10^ However, the neurobiological mechanism that underlies the spreading of Aβ action along a specific circuitry has not been elucidated yet.

Extracellular vesicles (EVs) are involved in neuron–neuron communication and glia-neuron communication. In neurodegenerative diseases, EVs act as carriers of pathogenic proteins, including Aβ, over large distances. Therefore, an intriguing hypothesis is that EVs could be implicated in the progression of synaptic dysfunction between connected regions. In a previous work, we demonstrated that a single injection of large microglial-derived EVs carrying Aβ (Aβ-EVs) in the lateral entorhinal cortex (LEC) is capable of inhibiting long-term potentiation (LTP), first in the vicinity of the injection site, i.e. superficial layer of LEC, and, at a later time point, also in its main target region, the dentate gyrus of the hippocampus. Moreover, *in vitro* investigation on primary cell cultures revealed that Aβ-EVs can move along neurites after their precise positioning at the neuron surface by optical tweezers,^11^ while inhibition of Aβ-EVs movement by pre-treatment of EVs with Annexin-V reduced the spreading of LTP impairment along the entorhinal-hippocampal circuitry. These results suggest that the motion of EVs carrying Aβ at the neuronal surface is crucial for the initiation and propagation of synaptic dysfunction along the entorhinal-hippocampal circuit, that was originally described in a genetic Alzheimer’s disease mouse model.^2^ As a follow-up to that study, here we sought to investigate whether a single injection of Aβ-EVs into the LEC could trigger cortico-hippocampal hyperexcitability and behavioural abnormalities. To this aim, we performed chronic EEG recordings following Aβ-EVs injection, to assess their effect on network stability. Electrophysiological results were compared with those obtained in a transgenic Alzheimer’s disease model to strengthen the hypothesis that Aβ-EVs effects can resemble the EEG alterations observed in a more comprehensive model of cerebral amyloidosis. Then, we investigated whether the spreading of synaptic dysfunction and consequent network alterations were associated with progressive memory impairments using behavioural tasks that differently involve LEC and hippocampus. In particular, we used an LEC-associative dependent task, the novel Object-Place-Context Recognition Test (OPCRT) and a hippocampal-dependent task, the novel Object Recognition Test (ORT) in order to assess the progression of memory impairment over time.^12,13^

## Materials and methods

### Animals

We used 3- to 4-month-old male C57BL/6 mice (Charles River, Lecco, Italy) wild-type and transgenic mice overexpressing an alternatively spliced human *APP* (mhAPP) minigene that encodes hAPP695, hAPP751 and hAPP770 bearing mutations linked to familial Alzheimer’s disease (V717F, K670N/M671L) (*APPsweInd*, line J20).^14^ All experimental procedures involving animals followed the guidelines defined by the European legislation (Directive 2010/63/EU) and the Italian Legislation (LD no. 26/2014). The Organism Responsible for Animal Welfare (OPBA) of the National Research Council of Italy (CNR) Institute of Neuroscience in Milan-Pisa and the Italian Ministry of Health approved the study protocol (authorizations 2D46A.N.KBG. and 233/2019-PR 129/2000-A). Accordingly, every effort was made to minimize the number of animals used and their suffering. In particular, the approved protocols included *a priori* power analysis that was performed using the software G*Power.^15^ The number of animals was calculated for the ANOVA test in order to achieve a power (1 − β) of 0.8 with alpha = 0.05, with an effect size based on the variability of preliminary data in similar control experiments and in previous work^13,16^ and an estimated 10% drop-out of animals.

### Primary cultures and EV isolation

Mixed glial cultures were established from postnatal Days 1–2 (P1–P2) C57BL/6 mice of either sex (Charles River, Lecco, Italy) and EVs were isolated as in Gabrielli *et al*.^11^ Briefly, Aβ(1–42)-treated microglia and control cells were washed and exposed to 1 mM ATP (Merck, Darmstadt, Germany) for 1 h in Krebs-Ringer’s HEPES solution (KRH) (in mM, 125 NaCl, 5 KCl, 1.2 MgSO_4_, 1.2 KH_2_PO, 2 CaCl_2_, 6 d-glucose, 25 HEPES/NaOH, pH 7.4). Conditioned KRH was collected and pre-cleared from cells and debris by centrifugation at 300*g* for 10 min, twice. Large-EV-enriched fraction was obtained by centrifuging the supernatant at 10 000*g* for 30 min at 4°C. EVs were used immediately after isolation.

### Aβ treatment

Microglia primary cultures were exposed to 2 μM Aβ 1–42 (cat. AS-20276, AnaSpec, Eurogentec, Liegi, Belgium) in a prevalent aggregated form for 20 h, as in our previous work showing that microglia is able to re-secrete Aβ in a soluble form in association with large EVs after internalization.^17^

The injected Aβ peptide was prepared as previously described,^11,18^ in order to obtain a mixture enriched in Aβ(1–42) oligomers. Briefly, the lyophilized synthetic Aβ 1–42 was suspended in 100% 1,1,1,3,3,3-hexafluoro-2-propanol (HFIP; Sigma-Aldrich), and HFIP was allowed to evaporate. The resulting peptide films were stored at −20°C. Twenty-four hours prior to use, the aliquots were added to DMSO (Sigma-Aldrich, MO, USA) and sonicated for 10 min. Oligomeric Aβ 1–42 was obtained by diluting an aliquot of Aβ 1–42/DMSO solution with a small volume of ACSF solution, vortexing for 30 s, and then incubating at 4°C for 16 h. Before using, oligomeric Aβ was added to ACSF to obtain 100 nM concentration.

### Annexin-V treatment

Large Aβ-EV pellet was resuspended in 300 μl KRH and Annexin-V (A7810, Merck, Darmstadt, Germany) was added at an active concentration of 8.4 μg/ml for 30 min on a low-speed wheel at room temperature. Subsequently, 1 ml KRH was added to the sample to dilute the unbound molecule and EVs were re-pelleted at 16 100*g* for 90 min at 4°C. Annexin V cloaks phosphatidyl serine (PS) residues, externalized on the surface of large EVs, strengthening cell membrane–EVs interaction.^11^

### Surgical procedures

Mice were deeply anaesthetized using an intraperitoneal injection of Zoletil 100 (zolazepam and tiletamine, 1:1, 40 mg/kg; Laboratoire Virbac) and Xilor (xilazine 2%, 10 mg/kg; Bio98). After the tail pinch reflex disappearance, mice were positioned in a stereotaxic apparatus, the scalp was shaved and a midline incision was made. Membranes of connective tissues covering the skull were removed to show cranial sutures, then two burr holes were drilled. A third hole was drilled to position a screw that was then connected to the ground. Bipolar electrodes were implanted in the parietal cortex (at the epidural level) and in the dentate gyrus of the hippocampus (DG, AP −2.2, ML +1.3 from the Bregma, DV +1.9 from the dura mater). A reference electrode was placed in correspondence of the cerebellum. All the electrodes were connected to a multipin socket and secured to the skull by acrylic dental cement. A 26 G guide cannula was then inserted at EC level (AP: −3.8; ML: + 4.0; DV: + 4) as a means for later injection of the different treatments in EC (as in Gabrielli *et al*.).^11^ Finally, a dental cement was used to fix the cannula and screws to the skull, and the scalp was sutured. In the immediate postoperative period, animals were housed in single cages located in a silent recovery area maintained at a temperature higher than that of housing (27–30°C) and then checked daily to monitor their well-being.

### In vivo electrophysiology and EEG analysis

**Figure 1:**
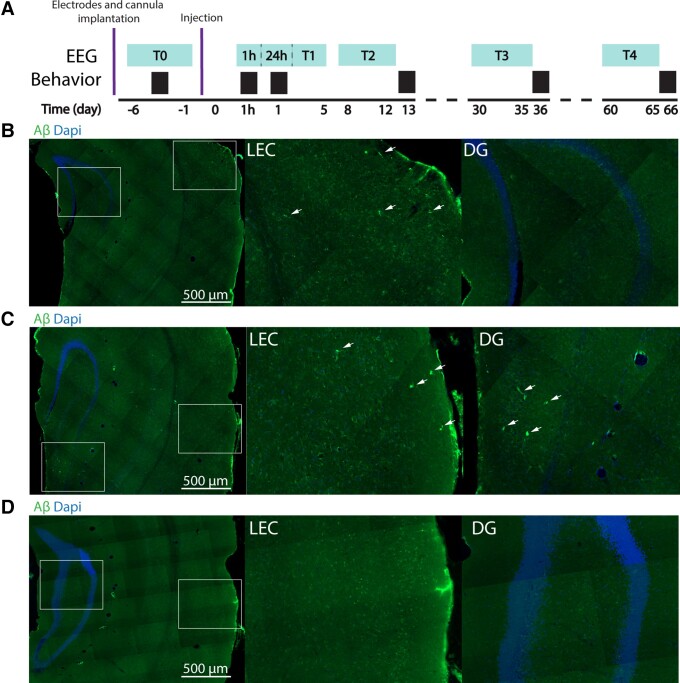
**Aβ detection along the entorhinal–hippocampal pathway following Aβ-EVs injection in the LEC.** (**A**) Experimental timeline indicating the time points for EEG recordings (light blue) and behavioural tests (black) following electrodes and cannula implantation and the time point of Aβ-EVs injection. (**B**) Aβ staining (in green) and DAPI staining (in blue) in the LEC and DG 1 h following the injection of Aβ-EVs in the LEC, showing the localization of Aβ at the level of the LEC. (**C**) Aβ staining (in green) and DAPI staining (in blue) 24 h after Aβ-EVs injection in the LEC show the presence of Aβ both at the level of the LEC and the DG. (**D**) No traces of Aβ are detected 2 months after Aβ-EVs injection. Aβ staining (in green) and DAPI staining (in blue).

One week after surgery, all mice were recorded for 2–4 h daily during a baseline period of five consecutive days in freely moving conditions. Then, an injecting needle was inserted through the guide cannula, 4 mm below the dura, and 1μl of EVs [0.25 × 108/μl in artificial CSF (ACSF)] was slowly introduced. Thus, Aβ-EVs (from microglia exposed to Aβ), CTRL-EVs (from microglia not exposed to Aβ), Aβ-EVs pre-coated with Annexin V, soluble oligomeric Aβ(1–42) alone (100 nM) or vehicle (ACSF) were administered at the EC level. ACSF composition was the following (in mM): 119 NaCl, 2.5 KCl, 2 CaCl_2_, 1.2 MgSO_4_, 1 NaH_2_PO_4_, 6.2 NaHCO_3_, 10 glucose, 10 HEPES. The pipette remained in place at the injection site for 2 min before slow removal to allow diffusion. Subsequent sessions of EEG recordings were performed at 24 h, 1 week, 1 and 2 months after injection. At each time point, the recording session was carried out 2–4 h daily for 5 consecutive days (Fig. 1A). Care was taken to record each animal at the same time of the day. Signals were recorded by a headstage (EXT-mini-2D, NPI electronic, Germany) amplified (10 000 fold) and band-pass filtered (0.3–100 Hz, EXT- 02F amplifier NPI electronic, Germany), digitized (sampling rate = 100 Hz, National Instruments card) and conveyed to a computer for storage and analysis. The recordings were performed using custom-made bipolar electrodes formed of two twisted enamel-insulated nichrome wires (120 μm in diameter) and the detection of seizures was performed with custom software written in LabView.^16^ The program first identifies spikes in the EEG using a voltage threshold set to 4.5 times the standard deviation of the EEG signal (determined in a period devoid of spike activity). To evaluate epileptiform activity, spike clusters lasting for more than 4 s were considered spontaneous recurrent seizures (SRS), while clusters lasting <4 s and isolated spikes were considered interictal events. For each recording session, the frequency and duration of SRS and interictal clusters, the number of single spikes and the total time spent in epileptiform activity (calculated by summing up the duration of either ictal or interictal episodes) have been determined. To evaluate changes in hippocampal and cortical excitability, we performed power spectral analysis using a custom-made MATLAB app. Spectral analysis was based on the multi-taper function of Chronux,^19^ a toolbox for MATLAB (The MathWorks, Natick, MA, USA). Multitaper analysis works by creating multiple tapers, therefore by applying windows to the data before computing the Fourier transform and averaging their spectra. The multitaper function allows using different smooth tapers to extract multiple independent projections (tapered data) from the noisy data, compute the single taper spectra and average them to create the multitaper spectrum. This method attenuates edge artefacts at the beginning and at the end of the epoch, thereby reducing the narrowband bias (that determines the frequency resolution) and the broadband bias (that corresponds to the spectral leakage). The frequency bands considered were Delta (0.1–4 Hz), Theta (4.1–8 Hz), Alpha (8.1–13 Hz), Beta (13.1–30 Hz) and Gamma (30.1–50 Hz).

*Sharp-wave discharges* (SWDs) are generalized epileptic-like activity and EEG data were analysed to screen out these events from cortical and hippocampal recordings. Raw EEG data were band-pass filtered between 7 and 23 Hz, an envelope was calculated by taking absolute values of Hilbert-transformed filtered data; a positive threshold was set above the envelope mean (absolute value of the Hilbert transform) and the two crossing points between the envelope and the threshold marked the range of a candidate SWD event.^20^ Eye inspection was needed to accept candidate events that showed at least three peaks over the threshold.

### Immunofluorescence analysis of Aβ

Mice were anaesthetized with 20% urethane (0.1 ml/100 g body weight, Sigma) by intraperitoneal injection and perfused via intracardiac infusion with PBS and then 4% paraformaldehyde (PFA, w/vol, dissolved in PBS). Brains were quickly removed and post-fixed overnight in PFA at 4°C, then transferred to 30% sucrose (w/vol) solution. Fifty micrometre coronal sections were cut on a freezing microtome (Leica) and free-floating sections were processed for immunofluorescence. The sections were incubated for 1 h in a blocking solution containing 5% normal goat serum (vol/vol) and 0.5% Triton X-100 (vol/vol) in PBS and incubated overnight at 4°C with anti-Aβ (cat. no. SIG-39300, BioLegend) diluted 1:1000 in PBS with 1% BSA (w/vol), normal goat serum 1% (vol/vol) and 0.1% Triton X-100 (vol/vol). Sections were then washed with PBS and incubated for 2 h at 22–24°C with Alexa Fluor 488-conjugated secondary antibody (cat. no. A-11001, ThermoFisher Scientific) which was added at a dilution of 1:500 in the same solution as the primary antibody. Sections were washed three times with PBS and mounted on slides, then they were air-dried and coverslipped with Vectashield mounting medium (cat. H-1000, Vector Laboratories). Imaging was performed on an Axio Imager Z2 microscope (Carl Zeiss) and multichannel images were produced with ApoTome 2 using an EC Plan-NEOFLUAR 10×/0.5 objective.

### Behavioural tests

The behavioural testing was performed within a 60 cm square box with 40 cm high walls as previously described.^13^ Following 1 week of extensive handling to habituate the mice to the experimenter, mice were individually habituated to the ‘white’ context (3 days) for 1 h each day. Behavioural testing proceeded in sequential stages for two consecutive days for either the novel object place/context (OPCRT) or the novel object (ORT) recognition test (see Supplementary Fig. 3).

### Statistical analysis

For all data acquisition, experimenters were blinded with respect to the treatment of mice. All data were analysed using GraphPad. For EEG recordings, after normality tests (D’Agostino–Pearson, Anderson–Darling, Shapiro–Wilk and Kolmogorov–Smirnov tests), differences between means were analysed by two-tailed Student’s *t*-test, unpaired and/or non-parametric Mann–Whitney test for non-normally distributed data. Also, two-way ANOVA was used followed by the Tukey test for multiple comparisons or the Kruskal–Wallis test followed by the Dunn test for non-normally distributed data.

For behavioural tests, one-way ANOVA was applied to determine the differences in average discrimination indices and exploration rates in the test phase for each recognition task. One-sample *t*-tests were also used to determine whether the average discrimination index for each group was different from chance (hypothesized mean = 0). Differences were considered significant when *P* < 0.05.

## Results

### Acute injection of Aβ-EVs induces a persistent aberrant excitatory activity

Microglial primary cultures were exposed to synthetic Aβ (2 µM for 20 h) and isolated by differential centrifugation after 1 mM ATP stimulation. The Aβ-EVs have been deeply characterized according to MISEV2018 guidelines^21^ by western blot, CONAN, TRPS and cryo-electron microscopy, both in our previous and in the present work^11^ (Supplementary Fig. 1).

We administered 1 μl Aβ-EVs (0.25 × 10^8^EV/μl in ACSF) through a guide cannula previously implanted in the LEC of adult C57BL/6J mice. Immunofluorescence analysis performed in WT mice injected with Aβ-EVs revealed that 1 h following the injection, Aβ peptide could be detected only at the level of the LEC (Fig. 1B), while 24 h later, a visible amount of the peptide was also observed in the dentate gyrus (Fig. 1C), suggesting that the Aβ initially present in the LEC propagated to the hippocampus in a time window of 24 h. The Aβ immunostaining became undetectable at 2 months following the injection (Fig. 1D). We confirmed that Annexin V affected the propagation of Aβ-EVs, as no signal was detected in DG at 24 after the injection (see Supplementary Fig. 2). Moreover, in control experiments, when Aβ alone was injected, the staining was confined to the EC; indeed, no signal was detected at the level of the hippocampus, even at 24 h after the injection. A quantification of the immunofluorescence detection of Aβ is reported in Supplementary Fig. 2.

**Figure 2:**
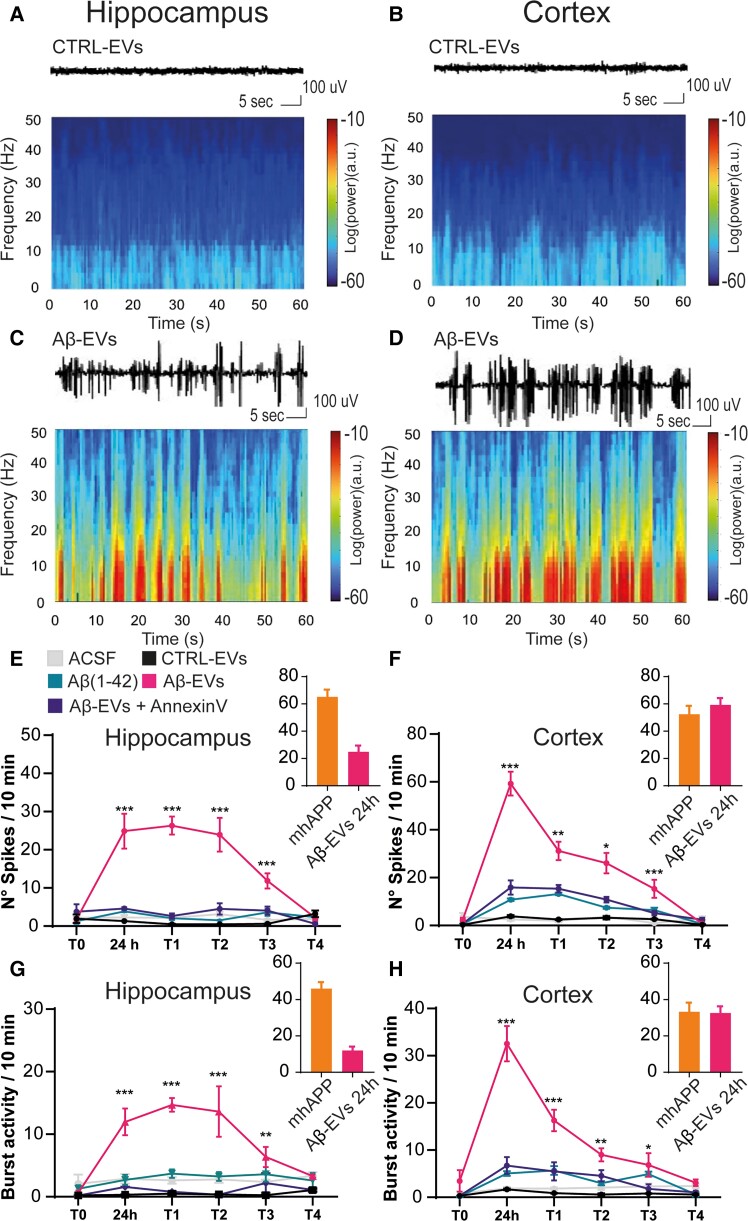
**Acute injection of Aβ-EVs induces a persistent aberrant excitatory activity.** (**A–D**) Example of raw traces and relative spectrograms of a control EVs-injected mouse (**A and C**) and a mouse injected with Aβ-EVs (**B and D**). (**E**) Total number of spikes in 10 min of recording in the hippocampus at different time points following Aβ-EVs injection in the LEC. Aβ-EVs-injected animals (pink line) showed a significant increase in spike frequency in the hippocampus compared with CTRL-EVs-injected mice (black line), either at 24 h (24.88 ± 4.58 spikes/10 min, *n* = 5 Aβ-EVs versus 1.30 ± 0.06 spikes/10 min, *n* = 4, *P* < 0.001 CTRL-EVs), 1 week (26.32 ± 2.35, *n* = 5 versus 0.49 ± 0.10, *n* = 4, *P* < 0.001), 2 weeks (23.92 ± 4.42, *n* = 5 versus 0.46 ± 0.06, *n* = 4, *P* < 0.001) or 1 month (11.84 ± 1.97, *n* = 5 versus 0.58 ± 0.12, *n* = 4, *P* < 0.001) and similar results were observed for ACSF-injected mice (24 h: 2.26 ± 0.65, *n* = 8 mice, *P* < 0.001; 1 week: 2.14 ± 0.47, *n* = 8 mice, *P* < 0.001; 2 weeks: 3.16 ± 0.61, *n* = 8 mice, *P* < 0.001; 1 month: 1.60 ± 0.27, *n* = 8 mice, *P* < 0.001). Aβ-EVs + Annexin-V-injected animals (violet line) had a significant lower number of spikes compared with Aβ-EVs-injected mice (pink line) either at 24 h (4.62 ± 0.32, *n* = 4 versus 24.90 ± 4.58, *n* = 5, *P* < 0.001), 1 week (2.63 ± 0.69, *n* = 4 versus 26.32 ± 2.35, *n* = 5, in Aβ-EVs + Annexin-V and Aβ-EVs, respectively; *P* < 0.001) or 2 weeks (4.5 ± 1.50, *n* = 4 versus 23.92 ± 4.42, *n* = 5, in Aβ-EVs + Annexin-V and Aβ-EVs, respectively; *P* < 0.001). No aberrant activity was detected in mice injected with Aβ(1–42) alone (green line, *n* = 4, *P* < 0.001 versus Aβ-EVs). Asterisks (***P < 0.001) indicate Tukey’s *post hoc* significance versus CTRL-EVs. (**F**) Total number of spikes in 10 min for cortical epidural recordings. Aβ-EVs-injected animals (pink line) showed a significant increase in spike frequency in the cortex compared with CTRL-EVs-injected animals (black line) either at 24 h (59.25 ± 4.99, *n* = 5 versus 3.86 ± 0.49, *n* = 4, *P* < 0.001), 1 week (31.15 ± 3.84, *n* = 5 versus 2.48 ± 0.25, *n* = 4, *P* < 0.001), 2 weeks (26.04 ± 4.28, *n* = 4 versus 3.21 ± 0.70, *n* = 4, *P* < 0.001) or 1 month after the injection (15.32 ± 3.76, *n* = 5 versus 2.62 ± 0.19, *n* = 4, *P* < 0.001) and similar results were observed in ACSF-injected mice (24 h: 2.5 ± 0.61, *n* = 8, *P* < 0.001; 1 week: 1.99 ± 0.61, *n* = 8, *P* < 0.001; 2 weeks: 3.7 ± 0.60, *n* = 8, *P* < 0.001; 1 month: 0.97 ± 0.25, *n* = 8, *P* < 0.001). Aβ-EVs + Annexin-V-injected animals (violet line) had a significant lower number of spikes compared with Aβ-EVs-injected mice (pink line) either at 24 h (15.88 ± 2.95, *n* = 4 Aβ-EVs + Annexin-V versus 59.26 ± 4.99, *n* = 5 Aβ-EVs, *P* < 0.001), 1 week (15.37 ± 0.15, *n* = 4 versus 31.15 ± 3.84, *n* = 5, in Aβ-EVs + Annexin-V and Aβ-EVs, respectively; *P* < 0.001) or 2 weeks (10.82 ± 1.17, *n* = 4 versus 26.04 ± 4.28, *n* = 4, in Aβ-EVs *P* < 0.001). No aberrant activity was detected in mice injected with Aβ(1–42) alone (green line, *n* = 4, *P* < 0.001 versus Aβ-EVs). (**G**) Bursting activity in the hippocampus. Recordings showed a significant increase in time spent in bursting activity in mice injected with Aβ-EVs (pink line) with respect to CTRL-EVs (black line) either at 24 h (11.97 ± 2.13, *n* = 5 Aβ-EVs versus 0.32 ± 0.07, *n* = 4, CTRL-EVs *P* < 0.001), 1 week (14.69 ± 1.09, *n* = 5 versus 0.48 ± 0.20, *n* = 4, *P* < 0.001), 2 weeks (13.62 ± 4.04, *n* = 5 versus 0.35 ± 0.05, *n* = 4, *P* < 0.001) or 1 month after the injection (6.35 ± 1.61, *n* = 5 versus 0.24 ± 0.07, *n* = 4, *P* < 0.01) and similar results were observed in ACSF-injected mice (24 h: 2.87 ± 0.75, *n* = 8, *P* < 0.001; 1 week: 2.6 ± 0.51, *n* = 8, *P* < 0.001; 2 weeks: 2.76 ± 0.54, *n* = 8, *P* < 0.001). Pre-treatment of Aβ-EVs with Annexin-V (violet line) was able to significantly reduce hippocampal bursting activity either at 24 h (Aβ-EVs with Annexin-V versus Aβ-EVs, *n* = 4 *P* < 0.001), 1 week (*n* = 4, *P* < 0.001) or 2 weeks (*n* = 4, *P* < 0.001). No aberrant activity was detected in mice injected with Aβ(1–42) alone (green line, *n* = 4, *P* < 0.001 versus Aβ-EVs). Asterisks (**P < 0.01, ***P < 0.001) indicate Tukey’s *post hoc* significance versus CTRL-EVs. (**H**) Bursting activity at the cortical epidural level. Recordings showed a significant increase in time spent in bursting activity in mice injected with Aβ-EVs (pink line) with respect to CTRL-EVs (black line) either at 24 h (32.54 ± 3.73, *n* = 5 versus 1.69 ± 0.17, *n* = 4, *P* < 0.001), 1 week (16.29 ± 2.27, *n* = 5 versus 0.87 ± 0.08, *n* = 4, *P* < 0.001), 2 weeks (9.00 ± 1.39, *n* = 5 versus 0.59 ± 0.26, *n* = 4, *P* < 0.01) or 1 month (6.85 ± 2.51, *n* = 5 versus 0.81 ± 0.05, *n* = 4, *P* < 0.05) and similar results were observed in ACSF-injected mice (24 h: 1.88 ± 0.35, *n* = 8, *P* < 0.001; 1 week: 1.86 ± 0.23, *n* = 8, *P* < 0.001; 2 weeks: 2.06 ± 0.48, *n* = 8, *P* < 0.001; 1 month: 2.33 ± 0.60, *n* = 8, *P* < 0.05). Pre-treatment of Aβ-EVs with Annexin-V (violet line) was able to significantly reduce cortical bursting activity either at 24 h (*n* = 4, *P* < 0.001) and 1 week (*n* = 4, *P* < 0.001). No aberrant activity was detected in mice injected with Aβ(1–42) alone (green line, *n* = 4, *P* < 0.001 versus Aβ-EVs). Asterisks (**P < 0.01, ***P < 0.001) indicate Tukey’s *post hoc* significance versus CTRL-EVs. Tukey’s *post hoc* significance versus all the other groups. The histograms inserted in (**E**–**H**) represent the values of the mhAPP group versus Aβ-EVs group at 24 h. Two-way ANOVA and Tukey’s test for multiple comparisons, Mann–Whitney test for mhAPP versus Aβ-EVs comparisons. Data are shown as mean ± SEM. CTRL-EVs, black line; Aβ-EVs, pink line; Aβ(1–42), green line; Aβ-EVs + Annexin-V, violet line.

To evaluate the effect of Aβ-EVs injections on neuronal activity, mice were subjected to longitudinal EEG recordings (Fig. 1A). In Fig. 2, the effect of Aβ-EVs on EEG recording neural activity is reported as representative traces and spectrograms. When compared with mice injected with CTRL-EVs (with EVs derived from microglia not exposed to Aβ), Aβ-EVs-injected animals exhibited epileptiform-like activity both in the hippocampus and at the cortical level (Fig. 2A–D). Quantification of spikes demonstrates increased excitability in the C57BL/6J mice after the Aβ-EVs injections in the EC, either at the level of the cortex or hippocampus. In particular, in the hippocampus, the Aβ-EVs-treated group showed a significant increase in the spike frequency 24 h after the injection compared with Ctrl. EEG recordings performed at later time points revealed persistence of hyperactivity as assessed at 1 week and at 2 weeks; which then rapidly declines 1 month after the injection of Aβ-EVs, and is completely lost at 2 months (Aβ-EVs pink line, CTRL-EVs black line Fig. 2E).

Similarly, in the brain cortex, Aβ-EVs-injected mice showed increased spike frequency compared with controls (ACSF and Ctrl-EVs groups) at 24 h after the injection. A significant increase was present at 1, 2 weeks and 1 month after the injection (Fig. 2F).

Our previous experiments demonstrated that limiting the movement of EVs by pre-treatment with Annexin-V was capable of reducing the spread of synaptic dysfunction from the LEC to the hippocampus.^11^ We performed EEG recordings in mice injected with Aβ-EVs pre-treated with 8.4 μg/ml of Annexin-V. The control treatment group with Annexin V alone was not applicable and anyway uninformative. Indeed, direct injection of Annexin V alone in the EC as a control would bind PS exposed on cells (e.g. apoptotic cells) and synapses (non-functional synapses) interfering with their normal removal by microglial cells, with no meaning for the current study. Also, our previous and present work revealed that control experiment using EVs alone had no significant effect and the result is comparable to control experiment (ACSF group). For this reason, we considered not to perform the EVs precoated with the Annexin V group, which would be uninformative. As reported in Fig. 2E and F (Aβ-EVs + Annexin-V violet line), the Aβ-EVs + Annexin-V group showed a significant reduction of hyperactivity with respect to mice injected with Aβ-EVs alone. Twenty-four hours after the injection Aβ-EVs + Annexin-V mice had a significant lower number of spikes with respect to Aβ-EVs in the hippocampus (Fig. 2E) and cortex (Fig. 2F). The same happened 1 and 2 weeks after injection of Annexin-V-coated Aβ-EVs.

The increased excitability of the network was further confirmed by analysing the bursting activity. Recordings showed a significant increase in time spent in bursting activity at 24 h in mice injected with Aβ-EVs (pink line) with respect to Ctrl-EVs (black line), either in the hippocampus or the cortex (Fig. 2G and H). The time course of bursting activity was consistent with that of the increase in the number of spikes as it was maintained for 2 weeks and 1 month after the injection, while rapidly normalizes at later time points (Fig. 2G and H). In agreement with what was described for total spikes number, pre-treatment of Aβ-EVs with Annexin-V (violet line) was able to significantly reduce hippocampal and cortical bursting activity (Fig. 2G and H). As a control experiment, we injected in the LEC synthetic oligomeric Aβ(1–42) peptide (100 nM) that was prepared as previously reported.^11^ No aberrant activity was detected in mice injected with Aβ(1–42) alone, suggesting that the association of Aβ with EVs is required for the initiation of cortico-hippocampal network hyperexcitability (Fig. 2E–H). Moreover, to further validate our acute model of Aβ-EVs-dependent neurodegeneration, we compared the EEG recordings of injected mice to that of transgenic animals overexpressing mutant human APP (mhAPP, Swe/Ind J20 line),^14^ in which hyperexcitability has been previously demonstrated.^2^ In the insets of Fig. 2E–H, a comparable level of activity in the hippocampus and the cortex is reported between the two groups.

### Aβ-EVs injection induces specific power spectra alterations

There is extensive literature on EEG metrics in both Alzheimer’s disease patients and Alzheimer’s disease transgenic models reporting specific alterations in EEG oscillations as a possible disease biomarker.^22,23^ To examine EEG alterations in mice injected with Aβ-EVs, the total power was calculated in the 1–50 Hz band, 2 weeks after the injection, when a significantly increased activity is detected in either the cortex or hippocampus but the number of total spikes and bursting activity is not at their maximal peak (between 24 h and 1 week after the injection). We decided to analyse the total power after the time window of maximal spiking activity in order to minimize its effect on calculation.

**Figure 3:**
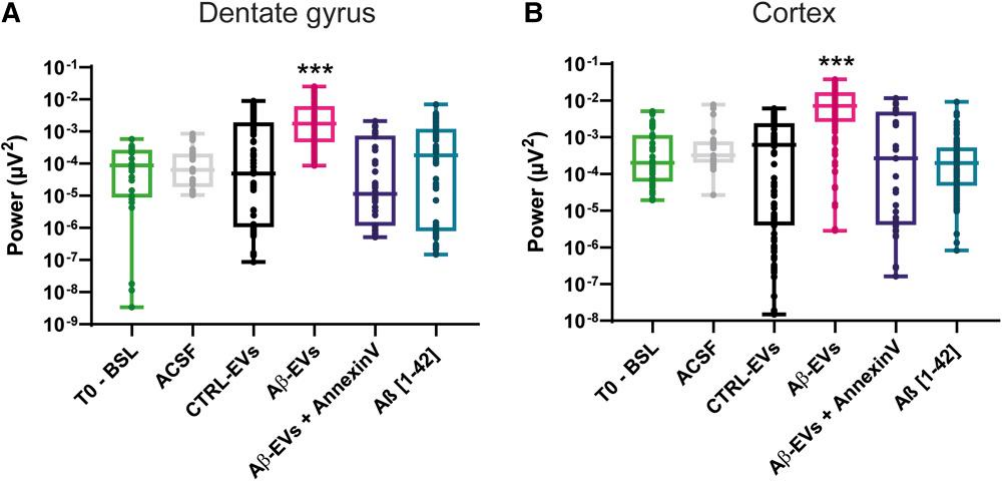
**Total power in the hippocampus and at the epidural level.** Total power (μV2) in 2 weeks of recordings after the injection according to the different groups. Two weeks after the injection represent the time window where the effect of Aβ-EVs is more prominent. (**A**) The analysis revealed a significantly increased power in the Aβ-EVs mice (pink) in the hippocampus compared with baseline recordings (light green, *P* < 0.001, *n* = 155 recordings, 5 mice for Aβ-EVs, *n* = 30 recordings, 5 mice for baseline), ACSF (light grey, *P* < 0.001, *n* = 33 recordings, 3 mice) and CTRL-EVs-injected animals (black, *P* < 0.001, *n* = 155 recordings, 4 mice). A significant reduction of the total power was observed in Aβ-EVs + Annexin-V-injected animals compared with Aβ-EVs (*P* < 0.001, *n* = 52 recordings, 4 mice) and in mice injected with Aβ(1–42) (*P* < 0.001, *n* = 112 recordings, 5 mice). (**B**) A significant increase in power was also observed in the cortex of animals injected with Aβ-EVs (pink) compared either with baseline recordings (light green, *P* < 0.001, *n* = 200 recordings, 5 mice for Aβ-EVs, *n* = 47 recordings, 5 mice for baseline), ACSF (light grey, *P* < 0.001, *n* = 29 recordings, 3 mice) or CTRL-EVs-injected animals (black, *P* < 0.001, *n* = 151 recordings, 4 mice). A significant reduction of the total power was observed in Aβ-EVs + Annexin-V-injected animals compared with Aβ-EVs (*P* < 0.001, *n* = 47 recordings, 4 mice) and a significant difference in the total power was observed in Aβ(1–42)-injected mice (*P* < 0.001, *n* = 127 recordings, 5 mice). Box plots show the median, 25th and 75th percentiles, and whiskers show min to max. T0, baseline recordings for all animals, Ctrl-EVs, Aβ-EVs, Aβ-EVs + Annexin-V. For all graphs, asterisks (***P < 0.001 versus all the groups) represent Dunn *post hoc* significance. The Kruskal–Wallis test followed by Dunn’s multiple comparisons test. T0-BSL, light grey; CTRL-EVs, black; Aβ-EVs, pink; Aβ-EVs + Annexin-V, violet.

Hippocampal recordings revealed a significant increase in the total power in the Aβ-EVs with respect to the baseline recordings before the injection (light grey bar) and to the Ctrl EVs (black bar) (Fig. 3A). Similarly, cortical recordings reported a significant increase in the total power in the Aβ-EVs (pink bar) with respect to the baseline recordings (light grey bar) and to the Ctrl-EVs (black bar) (Fig. 3B). However, pre-treatment with Annexin-V (violet bar) reduced the total power with respect to Aβ-EVs mice (pink bar), either in the hippocampus (Fig. 3A) or in the cortex (Fig. 3B).

**Figure 4:**
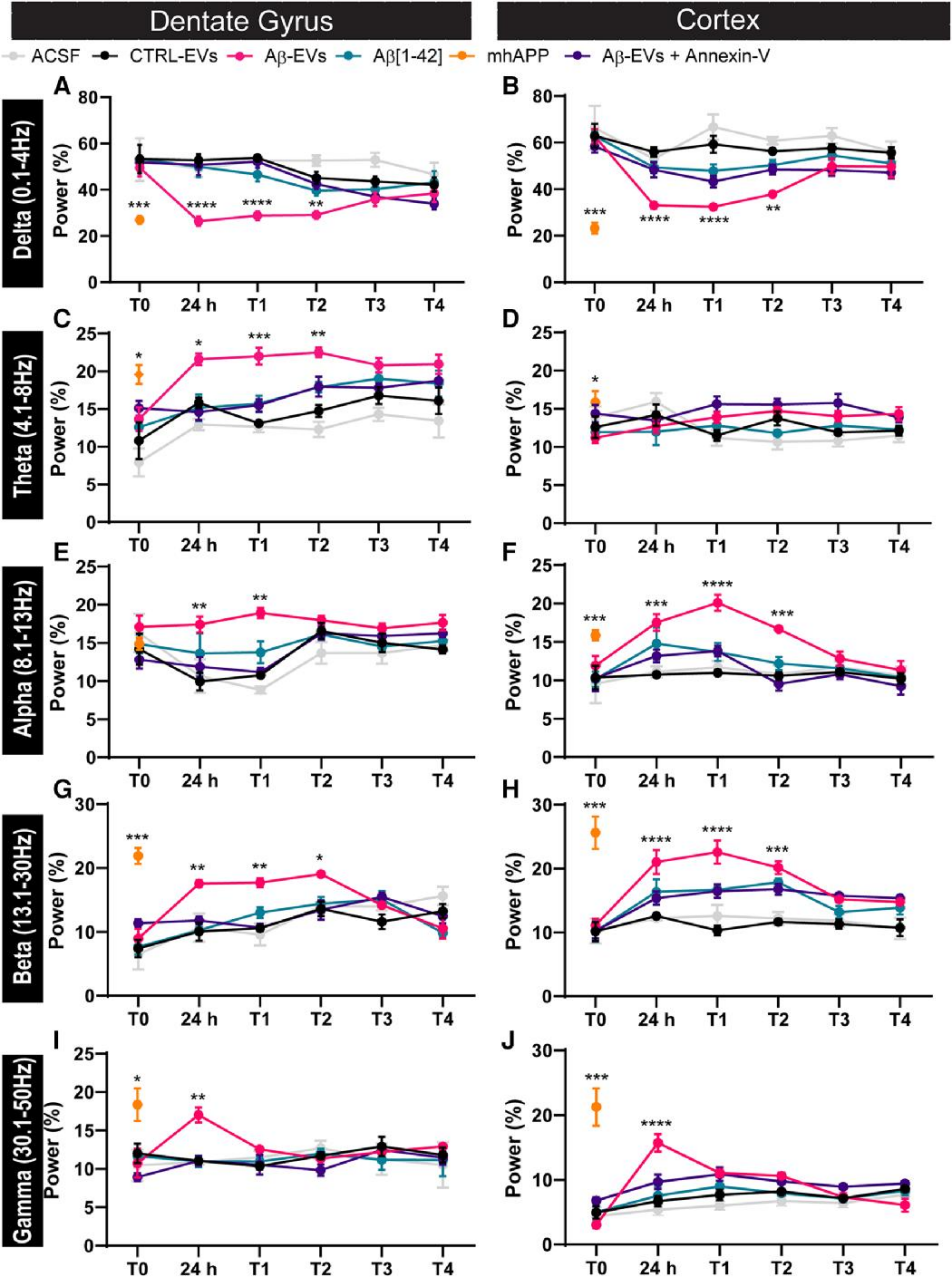
**Spectral analysis of the hippocampus and the cortex.** Chronic EEG recordings in freely moving mice at different time points. Power normalized on the total power of the 1–50 Hz band expressed in percentage (%) at different time points, divided in conventional frequency bands: (**A and B**) Delta (1–4 Hz), (**C** and **D**) Theta (4,1–8 Hz), (**E** and **F**) Alpha (8,1–13 Hz), (**G** and **H**) Beta (13,1–30 Hz) and (**I** and **J**) Gamma (30,1–50 Hz). Hippocampal recordings of Aβ-EVs mice (pink line) revealed statistically significant alterations from 24 h to 2 weeks after the injection in the Delta band (24 h: *P* < 0.001; 1 week: *P* < 0.001; 2 weeks: *P* < 0.01, *n* = 4 for CTRL-EVs, *n* = 5 for Aβ-EVs), Theta band (24 h: *P* < 0.05; 1 week: *P* < 0.001; 2 weeks: *P* < 0.01, *n* = 4 for CTRL-EVs, *n* = 5 for Aβ-EVs), Alpha band (24 h: *P* < 0.01; 1 week: *P* < 0.01, *n* = 4 for CTRL-EVs, *n* = 5 for Aβ-EVs), Beta band (24 h: *P* < 0.01; 1 week: *P* < 0.01; 2 weeks: *P* < 0.05, *n* = 4 for CTRL-EVs, *n* = 5 for Aβ-EVs) and Gamma band (24 h: *P* < 0.01, *n* = 4 for CTRL-EVs, *n* = 5 for Aβ-EVs) with respect to CTRL-EVs. A similar effect was observed in the cortex, in the Delta band (24 h: *P* < 0.001; 1 week: *P* < 0.001; 2 weeks: *P* < 0.01, *n* = 4 for CTRL-EVs, *n* = 5 for Aβ-EVs), Alpha band (24 h: *P* < 0.001; 1 week: *P* < 0.001; 2 weeks: *P* < 0.001, *n* = 4 for CTRL-EVs, *n* = 5 for Aβ-EVs), Beta band (24 h: *P* < 0.001; 1 week: *P* < 0.001; 2 weeks: *P* < 0.001, *n* = 4 for CTRL-EVs, *n* = 5 for Aβ-EVs), Gamma band (24 h: *P* < 0.001, *n* = 4 for CTRL-EVs, *n* = 5 for Aβ-EVs) (Fig. 4J) compared with CTRL-EVs. Data are shown as mean ± SEM. For all graphs, asterisks (***P < 0.001; **P < 0.01; *P < 0.05) represent Tukey *post hoc* significance versus other groups. Two-way ANOVA and Tukey’s test for multiple comparisons. ACSF, light grey; CTRL-EVs, black; Aβ-EVs, pink; Aβ(1–42), green; Aβ-EVs + Annexin-V, violet.

Moreover, when we performed spectral analysis, we found significant changes in the main frequency bands that were induced in the group of mice that were injected with Aβ-EVs (Fig. 4). In particular, hippocampal recordings of Aβ-EVs mice (pink line) revealed statistically significant alterations that started 24 h after the injection and lasted for 2 weeks in the Delta (0.1–4.0 Hz) band (Fig. 4A), Theta (4.1–8.0 Hz) band (Fig. 4C), Alpha (8.1–13.0 Hz) band (Fig. 4E), Beta (13.1–30.0 Hz) band (Fig. 4G) and Gamma (30.1–50.0 Hz) band (Fig. 4I) with respect to the controls (Ctrl-EVs black line and ACSF grey line); all these alterations reduced gradually starting from 1 month after the injection and are completely lost after 2 months (Fig. 4). A similar alteration was found in mhAPP mice (Fig. 4, orange line); in contrast, the time course analysis revealed no significant changes in controls and Aβ(1–42)-injected mice (green line). However, in agreement with what was reported for spikes count, the effect of Aβ-EVs on spectral analysis of the main frequency bands was significantly reduced by Annexin-V pre-treatment of Aβ-EVs, either in the hippocampus and cortex (Fig. 4 violet line). A similar effect on the power was observed at the cortical level. Indeed, Aβ-EVs mice (pink line) showed alterations in the Delta band (Fig. 4B), Alpha band (Fig. 4F), Beta band (Fig. 4H) and Gamma band with respect to the controls (Ctrl-EVs black line and ACSF grey line).

### Sharp wave discharges are detectable after Aβ-EVs injection

**Figure 5:**
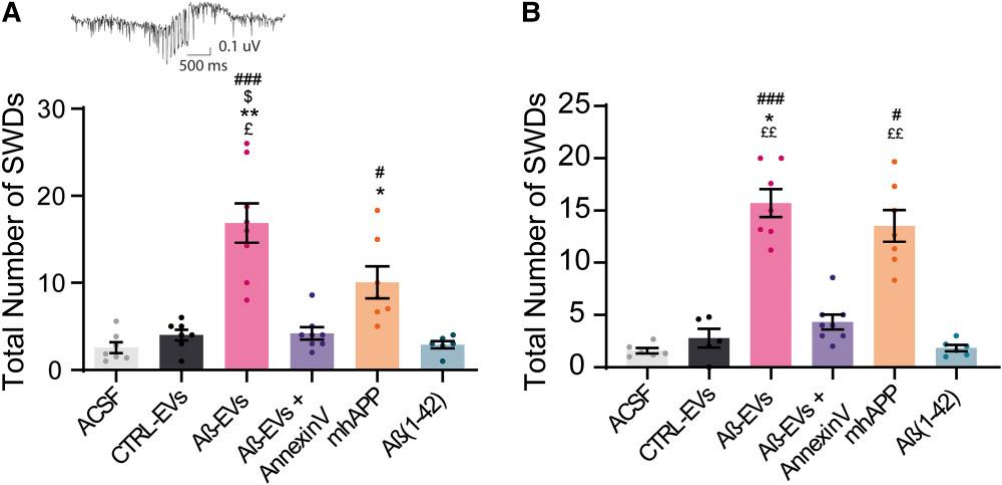
**Total number of SWDs in the hippocampus and at the epidural level.** (**A**) The analysis of total SWDs detected at the hippocampal level showed an increase in SWDs in the DG in mice injected with Aβ-EVs (pink bar; 16.88 ± 2.3, *n* = 7 mice) compared with mice injected with CTRL-EVs (4 ± 0.62, *n* = 7 mice, *P = 0.0395), ACSF (2.57 ± 0.62 *n* = 7 mice, ###*P* = 0.0002) or Aβ-EVs + Annexin-V (4.20 ± 0.70, *n* = 8 mice, $*P* = 0.025). No significant difference was observed between Aβ-EVs-injected animals and mhAPP animals (orange bar). Aβ(1–42)-injected mice showed a lower number of SWDs compared either with Aβ-EVs-injected animals (2.9 ± 0.42, *n* = 6 mice Aβ(1–42) versus 16.88 ± 2.3, *n* = 7 mice Aβ-EVs, ££*P* = 0.012) or mhAPP mice (10.05 ± 1.84, *n* = 7 mice mhAPP, #*P* = 0.0384). mhAPP mice were also significantly different from ACSF mice (10.05 ± 1.84, *n* = 7 mice mhAPP versus 2.57 ± 0.62, *n* = 7 mice ACSF, ***P = 0.0112). (**B**) SWD analysis at the cortical epidural level showed an increase in the total number of SWDs in mice injected with Aβ-EVs (14 ± 2.06, *n* = 7, pink bar) compared with mice injected with CTRL-EVs (2.78 ± 0.89, *n* = 5, *P = 0.0383), ACSF (1.58 ± 0.25, *n* = 6 ACSF, ###*P* < 0.001), Aβ-EVs + Annexin-V (4.32 ± 0.71, *n* = 8; $*P* = 0.025) and Aβ(1–42) (1.83 ± 0.30, *n* = 6, ££*P* = 0.0017). A higher number of SWDs was also observed in mhAPP compared with controls (13.52 ± 1.5, *n* = 7 mice mhAPP versus 1.58 ± 0.25, *n* = 6 ACSF, ***P = 0.0028) and Aβ(1–42) (1.83 ± 0.30, *n* = 6, ££*P* = 0.01). Data are reported as mean ± SEM. The Kruskal–Wallis test followed by Dunn’s multiple comparisons test.

The presence of sharp waves discharges (SWDs) is a typical feature of cortical network alterations.^20,24–26^ The analysis revealed discharge complexes both in hippocampal and in cortical recordings, that resembled classic SWDs in genetic rat models of absence epilepsy^27^ and correlates with discharge events found by Jin *et al*.^20^ The total number of SWDs in the hippocampus was quantified including pooled events from 24 h to 2 weeks after the injection (T2). The analysis revealed few SWDs in controls EVs and ACSF-treated groups, but a significantly higher number of events in the Aβ-EVs group (Fig. 5A). The presence of SWDs was also detected in the cortical recordings from the Aβ-EVs group with respect to controls (ACSF and Ctrl EVs, Fig. 5B). It is worth to notice that SWDs were also detected in mhAPP mice (Fig. 5).

In agreement with spike analysis, Aβ-EVs pre-treatment with Annexin-V reduced the number of SWDs—either in the hippocampus or in the cortex (Fig. 5A and B).

### Associative LEC-dependent memory is immediately affected 1 h after Aβ-EVs injection

**Figure 6:**
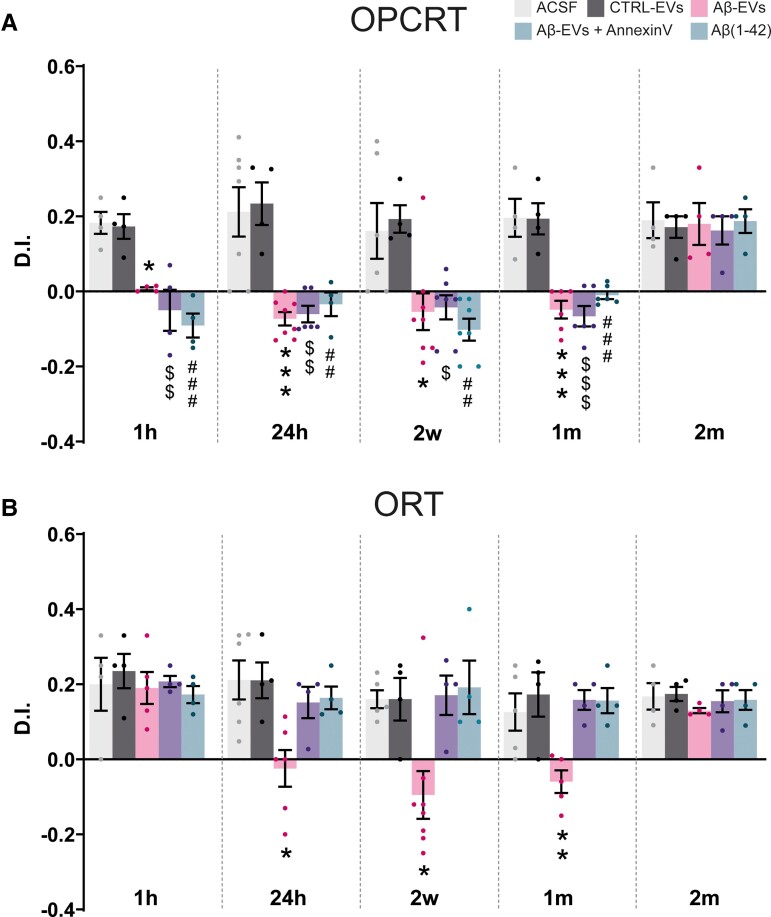
**Effects of Aβ-EVs injection on LEC-dependent (OPCRT) and hippocampal-dependent (ORT) behavioural paradigms.** (**A**) The average DI for Aβ-EVs mice was not significantly different by chance (*P* = 0.1833, *n* = 8 mice) at 1 h after the injection. The average DIs in the OPCRT were calculated for each group at six different time points: 1 h (0.007 ± 0.004, *n* = 4 mice Aβ-EVs versus 0.17 ± 0.03, *n* = 4 mice in CTRL-EVs *P < 0.05; −0.05 ± 0.05, *n* = 4 mice Aβ-EVs + Annexin-V versus 0.17 ± 0.03, *n* = 4 mice in CTRL-EVs, $$*P* < 0.01; 0.18 ± 0.03 *n* = 4 mice, of ACSF-injected mice versus −0.09 ± 0.03, *n* = 4 Aβ(1–42), ###*P* < 0.001), 24 h (−0.07 ± 0.02, *n* = 8 mice Aβ-EVs versus 0.23 ± 0.06, *n* = 4 CTRL-EVs, ***P < 0.001; −0.06 ± 0.02, *n* = 6 mice Aβ-EVs + Annexin-V versus 0.23 ± 0.06, *n* = 4 CTRL-EVs, $$*P* < 0.01; −0.03 ± 0.03, *n* = 4 mice Aβ(1-42) versus 0.21 ± 0.07, *n* = 7 mice ACSF, ##*P* < 0.01), 2 weeks (−0.05 ± 0.05, *n* = 8 mice Aβ-EVs versus 0.19 ± 0.07, *n* = 6 mice CTRL-EVs, *P < 0.05; −0.04 ± 0.03, *n* = 7 Aβ-EVs + Annexin-V versus 0.19 ± 0.04, *n* = 4 mice CTRL-EVs; −0.1 ± 0.03, *n* = 7 mice Aβ(1–42) versus 0.16 ± 0.07, *n* = 6 mice ACSF, $*P* < 0.05; ##*P* < 0.01), 1 month (−0.05 ± 0.02, *n* = 6 mice Aβ-EVs versus 0.19 ± 0.04, *n* = 4 mice CTRL-EVs, ***P < 0.001; −0.07 ± 0.03, *n* = 6 mice Aβ-EVs + Annexin-V versus 0.19 ± 0.04, *n* = 4 mice CTRL-EVs, $$$*P* < 0.001, −0.01 ± 0.01, *n* = 6 mice Aβ(1–42) versus 0.19 ± 0.05, *n* = 4 mice ACSF, ###*P* < 0.001) and 2 months after injection. Memory performance in Aβ-EVs-injected mice was significantly different from chance at 2 months after the injection (0.18 ± 0.06, *n* = 4, *P* = 0.0484). Data are reported as mean ± SEM. (**B**) One hour after the injection, the DI of Aβ-EVs-injected mice was significantly different from chance (0.19 ± 0.042, *n* = 5, *P* = 0.0111). The averaged DIs in the ORT were calculated for each group at six different time points: 1 h, 24 h (−0.02 ± 0.05, *n* = 6 mice Aβ-EVs versus 0.21 ± 0.05, *n* = 6 mice CTRL-EVs, *P < 0.05), 2 weeks (−0.09 ± 0.06, *n* = 8 mice Aβ-EVs versus 0.16 ± 0.06, *n* = 4 mice CTRL-EVs, *P < 0.05), 1 month (−0.06 ± 0.03, *n* = 5 mice Aβ-EVs versus 0.17 ± 0.06, *n* = 4 mice CTRL-EVs, **P < 0.01) and 2 months after injection. Data are reported as mean ± SEM. One-way ANOVA and Tukey’s multiple comparisons test. ACSF, grey; CTRL-EVs, black; Aβ(1–42), green; Aβ-EVs, pink; Aβ-EVs + Annexin-V, violet.

Given the progression of synaptic dysfunction caused by Aβ-EVs from EC to the hippocampus,^11^ we aimed at clarifying whether Aβ-EVs could differently affect associative memory, which is LEC dependent, and non-associative memory, which is LEC-independent but requires hippocampal function. To this aim, we used the non-associative ORT test and the associative OPCRT test (OPCRT), which was characterized as an LEC-dependent task.^12,13^ As shown in Fig. 6, we analysed ORT and OPCRT for each different group of mice. A separate group of mice was tested at each time point to avoid memory interference by test repetition. No differences were found between groups in exploratory and locomotor activities (see Supplementary Fig. 3).

The performance of Aβ-EVs-injected mice revealed an impairment in the ability to discriminate the novel object in relation to its position/context, 1 h after the injection in the EC, suggesting a memory deficit caused by a local effect. The average discrimination index (DI) for Aβ-EVs mice was not significantly different by chance and significantly different when compared with the control groups 1 h after the injection (Fig. 6A). In contrast, the ORT was not affected at this time point (Fig. 6B). The impairment in the OPCRT persisted even at a later time point. Indeed, the DI of Aβ-EVs-injected mice remains significantly lower compared with the CTRL-EVs-injected mice at 24 h, 2 weeks and 1 month after the injection. Memory performance recovered 2 months after the injection. A local effect in the LEC was confirmed in mice injected with Aβ(1–42) alone, which had significantly different DI from controls ACSF-injected mice 1, 24 h, 2 weeks and 1 month after the injection of Aβ(1–42) in the EC. After the treatment of Aβ-EVs with Annexin-V, the DI was significantly different from control EVs at all the following time points: 1, 24 h, 2 weeks and 1 month. However, in the group of mice that were tested 2 months after the injection, Annexin-V did not affect the memory process (Fig. 6A), and the DI was comparable to that of the other treatment groups. These results suggest that Aβ-EVs have a fast local effect when injected in the EC, which then lasts for ∼2 months; moreover, limiting EVs motion does not rescue the impairment in EC-dependent associative memory.

### Non-associative hippocampal- dependent memory starts to be affected 24 h after Aβ-EVs injection

As shown in Fig. 6B, we analysed the hippocampal-dependent ORT for each treatment group.

One hour after the injection of Aβ-EVs (Fig. 6B), the animals could discriminate between familiar and novel objects, the average DI was significantly different from chance and not significantly different compared with either ACSF-injected group or CTRL-EVs-injected group. The ORT memory was also not affected in synthetic Aβ(1–42)-injected animals, and in Aβ-EVs + Annexin-V treated mice. However, mice injected with Aβ-EVs showed a memory impairment and a significant difference in terms of DI compared with control mice at later time points, 24 h, 2 weeks and 1 month after the injection, suggesting a progression of the neuronal dysfunction along EC-hippocampal circuitry. However, the effect of Aβ-EVs on memory was not present in mice evaluated 2 months after the injection, and no significant difference was found between groups at this time point (Fig. 6B).

The progression of memory impairment was not present when mice were injected with Aβ(1–42) alone, showing no significant difference compared with controls 24 h after the injection, and at later time point: 2 weeks, 1 and 2 months after the injection.

Non-associative memory was not affected by Aβ-EVs in the presence of Annexin-V. The DI of this treatment group never differed from that of control EVs: 24 h after, 2 weeks after, 1 month after and 2 months after the injection at the level of EC. These findings indicate that Aβ can propagate its effect when is associated with EVs causing progressive memory impairment.

## Discussion

The exact mechanisms of Aβ trans-synaptic propagation are yet to be discovered; however, increasing evidence indicates that microglia EVs could be involved in the spreading of amyloid and subsequent synaptic dysfunction within synaptically connected regions. Indeed, EVs have an important role in transcellular signalling and they have been demonstrated to be able to transfer pathogenic proteins.^17,28–30^ In a previous work, we demonstrated that: (i) large microglial EVs carrying Aβ (Aβ-EVs) move at the axon surface *in vitro*^11^ and (ii) the stereotaxic injection of Aβ-EVs in the LEC of WT animals is sufficient to inhibit LTP in the vicinity of the injection site first, and then at the level of the dentate gyrus 24 h later, indicating propagation of Aβ-dependent synaptic dysfunction along the EC–hippocampal axis. In the present work, we show that a single Aβ-EVs injection at the level of the LEC induces EEG abnormalities of the cortico-hippocampal network, which can be observed until 1 month after the injection. In particular, Aβ-EVs-injected mice showed a significant increase in the total number of spikes and bursting activity both at the level of the EC and dentate gyrus at 24 h after the injection, that then persists up to a month later and is completely reversed at 2 months. This hyperexcitability recapitulated the abnormalities observed in the *APP* transgenic mouse model of Alzheimer’s disease (mhAPP Swe/Ind J20 line)^2,14^ that were used to demonstrate that the endogenous production and trans-synaptic progression of Aβ along the performant pathway to the DG induces a rearrangement of inhibitory inputs to the DG cells leading to aberrant network activity. It is plausible that a similar effect on excitatory/inhibitory balance at the level of DG can be temporarily obtained by the injection of Aβ EVs. We suggest that the action of Aβ could initiate a persistent change at the level of EC and DG circuitry. Indeed, we have previously demonstrated that Aβ-EVs treatment can modulate the plasticity of the intrinsic EC circuitry, an effect that was associated with changes in the excitability of EC layer II neurons as assessed by patch-clamp recordings. Similar alterations were then found in the DG cells at 24 h after the injection.^11^ Hyperexcitability was not observed when Aβ-EVs were pre-treated with Annexin-V, an inhibitor of extracellular EV motion along axons, suggesting that Aβ-EV motion at the neuronal surface along the EC–DG pathway is crucial for inducing network dysfunction. Interestingly, spectral analysis revealed a general increase in higher frequencies and a decrease in the power of lower frequencies in our EEG recordings, in agreement with other studies in transgenic Alzheimer’s disease mouse models.^31–37^ These changes gradually reduced starting at 1 month after injection, while hyperexcitability in terms of bursting activity and isolated spikes is completely reversed only after 2 months of injection. The most plausible explanation is that the spectral analysis of the main frequencies bands is inevitably affected by the initial robust bursting activity, especially the beta and gamma frequencies. Whether frequency-specific patterns of neural activity in Alzheimer’s disease are interpreted as transient bursts of isolated events rather than as rhythmically sustained oscillations is a highly debated matter,^38,39^ we suggest that our analysis is less likely to detect a significant change in the power of frequency bands when the bursting activity is progressively reducing. In Alzheimer’s disease patients, EEG analysis usually reports a reduction of oscillatory activity with increased power in the Delta and Theta bands and decreased power in the higher frequencies. Although at first glance the results in MCI/Alzheimer’s disease patients could appear in contrast with our findings, this difference could be explained by the fact that EEG patterns change according to the amyloid burden. Indeed, preclinical Alzheimer’s disease patients show increased higher frequency power and decreased lower frequency power during the awake state. However, when Aβ load exceeds a certain threshold, the trend reverts and correlates with the classical aforementioned Alzheimer’s disease metrics, due to the failure of compensatory mechanisms.^40^ This evidence might indicate that our acute model of neurodegeneration can indeed recapitulate the spectral abnormalities observed at the early stages of Alzheimer’s disease in preclinical patients. Moreover, spectral alterations were not observed in the presence of Annexin-V, further strengthening the hypothesis that inhibiting EVs movement can reduce the spreading of synaptic dysfunction within the entorhinal–hippocampal circuitry^11^ and prevent the onset of hyperexcitability. However, we want to point out that Annexin-V does not represent an appropriate tool to slow down Alzheimer’s disease progression. In fact, in spite of its effectiveness in blocking the propagation of Aβ-related synaptic dysfunction, it binds PS exposed on cells (e.g. apoptotic cells) and synapses (non-functional synapses), thus possibly interfering with their normal removal by microglial cells.

It is worth noticing that epileptic activity without visible convulsions is a common feature in Alzheimer’s disease and may contribute to the disease pathogenesis.^41^ Many patients with Alzheimer’s disease experience fluctuations in cognitive functions, such as transient episodes of amnestic wandering and disorientation, which probably derive from aberrant network activity.^42,43^ Subclinical epileptiform activity, specifically SWDs, were also previously reported in *APP/PS1* mice.^20^ We were able to isolate SWDs in our model of early Alzheimer’s disease pathology, induced by a single injection of Aβ-EVs. We observed an increase in the number of SWDs in Aβ-EVs injected mice, both in the cortex and hippocampus, with respect to the controls and animals injected with Aβ-EVs pre-treated with Annexin-V. The precise temporal pattern of neuronal dysfunction and circuitries involvement during the progression of the disease led us to hypothesize that the spreading of EVs along the EC–hippocampal circuit should initially impair EC-dependent memory and affect hippocampal-related memory only at later time points.

A growing body of evidence has linked the EC to the formation of episodic associative memories. Indeed, the medial and lateral subdivisions of EC are thought to integrate spatial and non-spatial contextual information during an experience in service of memory.^44^ Interestingly, LEC lesions have been shown to impair the ability to perform the OPCRT, an associative behavioural paradigm widely used to assess episodic-like memory in mice^12,45^ and, more recently, a specific subpopulation of the LEC superficial layers, the fan cells, has been shown to be crucial for the correct execution of the same behavioural task, further strengthening the hypothesis that the EC, and in particular its lateral subdivision, plays a key role in episodic memory.^46^ As expected and in line with our hypothesis, in Aβ-EVs-treated mice the LEC-dependent memory task OPCRT was impaired already 1 h after the injection and the same behavioural alterations were observed in mice injected with Aβ(1–42), suggesting that the presence of the amyloid peptide in the EC is sufficient to induce episodic-like memory impairment, as previously reported in transgenic models of cerebral amyloidosis.^13^ However, non-associative hippocampal-dependent object recognition memory remained preserved when mice were tested 1 h after the injection while it started to be impaired 24 h later, reflecting the progression of Aβ-induced synaptic dysfunction from the EC to the hippocampus at later time points. The reduction of EVs mobility by the Annexin-V coating treatment prevented the impairment of the hippocampal-dependent memory task, suggesting that EVs motion through EC-hippocampal connections is necessary for the progression of behavioural deficits. Importantly, the present findings suggest that the EVs-mediated spreading of Aβ along the EC-hippocampal circuit could represent a pathological mechanism for trans-synaptic progression of Aβ-induced neuronal dysfunction and behavioural alteration also in animals overexpressing mutant APP at the EC level.^4^

## Conclusion

Our study demonstrates that microglial-derived Aβ-EVs participate in the spreading of amyloid pathology along synaptically connected regions and that this can lead to abnormalities in network activity and memory performance that are reminiscent of the early stages of Alzheimer’s disease pathology. Our work also strengthens the hypothesis that EEG recordings hold great potential as a possible non-invasive biomarker for Alzheimer’s disease and suggests that our acute model of early Alzheimer’s disease pathology could be used in the future to test pharmacological treatments to target the early stage of the disease.

## Supplementary Material

fcad170_Supplementary_Data

## Data Availability

The data that support the findings of this study are available from the corresponding authors upon reasonable request.

## Abbreviations

aCSF =: artificial CSF
Aβ =: β-amyloid
APP =: amyloid precursor protein
BSA =: bovine serum albumin
CONAN =: colorimetric nanoplasmonic assay
Cryo-EM =: cryo-electron microscopy
DG =: dentate gyrus
DI =: discrimination index
DMSO =: dimethyl sulfoxide
EC =: entorhinal cortex
EVs =: extracellular vesicles
HFIP =: 1,1,1,33,3-hexafluoro-2-propanol
KRH =: Krebs-Ringer’s HEPES solution
LEC =: lateral entorhinal cortex
LTP =: long-term potentiation
MCI =: mild cognitive impairment
mhAPP =: mutant human APP transgenic mice
OPCRT =: novel object-place-context recognition test
ORT =: novel Object Recognition Test
PFA =: paraformaldehyde
PS =: phosphatidyl serine
PS1 =: presenilin 1
SRS =: spontaneous recurrent seizures
SWDs =: sharp waves discharges
TRPS =: Tunable Resistive Pulse Sensing technique
