# Supplementary material for: Microglial extracellular vesicles induce Alzheimer’s disease-related cortico-hippocampal network dysfunction
Source: Brain Commun. 2023 May 31;5(3):fcad170. doi: 10.1093/braincomms/fcad170 (PMC10243901; doi:10.1093/braincomms/fcad170)
Supplement: fcad170_Supplementary_Data [file fcad170_supplementary_data.pdf]

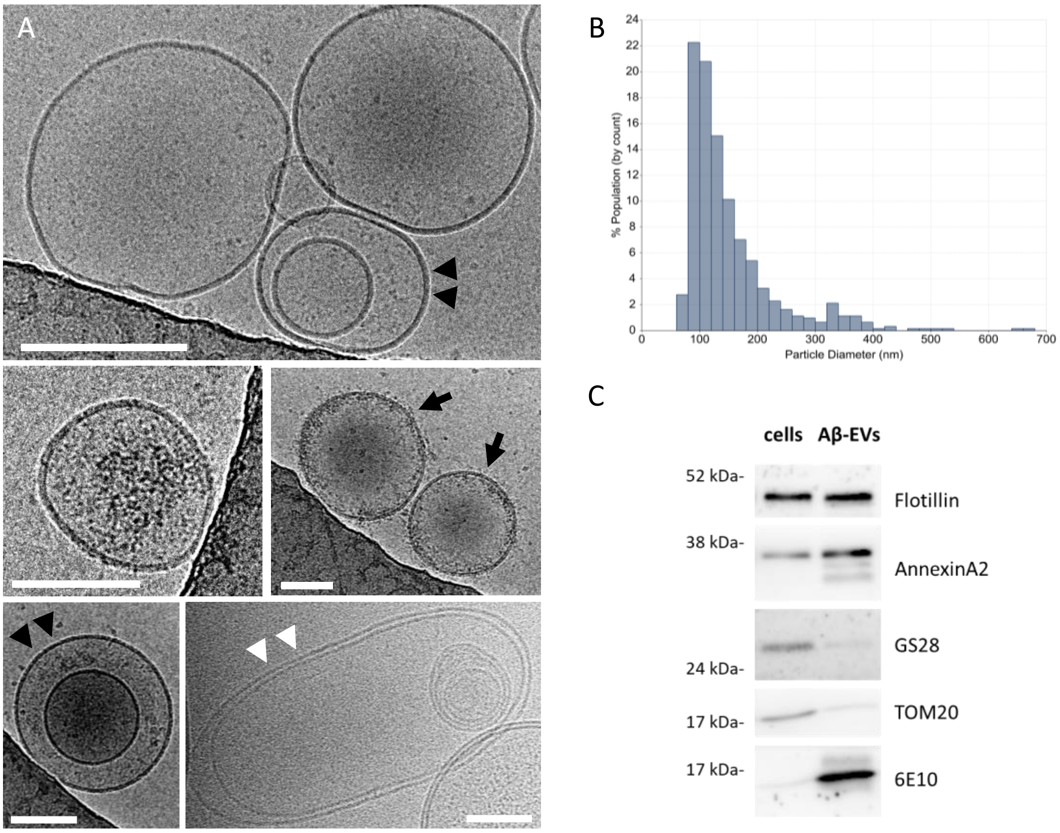

Supplementary Figure 1. A) Representative cryo-electron micrographs of Aβ-EVs. Microglial large EVs are heterogeneous in morphology: some of them present a rough surface (black arrows); most of them are rounded, some are multilamellar (black arrowheads), others tubular (white arrowheads). Scale bars: 100 nm. Freshly prepared large-Aβ EVs resuspended in saline were vitrified by applying a 3.5-μl droplet onto a holey carbon grids (Copper 300-mesh Quantifoil R2/1) glow discharged for 45 seconds at 40 mA using a GloQube system (Quorum Technologies, East Sussex, UK). After 60 seconds incubation, the grid was plunge-frozen in liquid ethane using a Vitrobot Mk IV (Thermo Fisher Scientific) operating at 4°C and 100% RH. Images of the vitrified specimen were acquired using a Talos Arctica transmission electron microscope (Thermo Fisher Scientific) operating at 200 kV and equipped with a Falcon 3EC direct electron detector (Thermo Fisher Scientific). **B)** The histogram shows the size distribution of Aβ-EVs obtained by TRPS analysis using qNano instrument (IZON) and NP300 nanopores (Izon, Christchurch, New Zealand). CPC200 calibration particles were used as standards. Samples and beads were recorded in PBS + 0.03% w/v Tween20. Data acquisition and analysis were performed on Izon Control Suite software (version V3.3). **C**) Aβ-EV Western Blot characterization shows the presence of the EV marker Flotillin, the enrichment of the large EV marker Annexin A2 and of Aβ (6E10), and the depletion of Golgi and mitochondria markers (GS28 and TOM20, respectively) in the Aβ-EV fraction compared to donor cell lysate (2 μg). Aβ is highly enriched in EVs compared with donor cells (about 20-fold change). Microglia were lysed with a buffer containing 0.29 M Sucrose, 10% SDS, 0.5 M Tris pH 6.8 and Halt Protease Inhibitor (1:100; cat.#78430, Thermo Fisher Scientific). A modified version of the Laemmli buffer was then added to a final 1× concentration (15% SDS, 575 mM sucrose, 325 mM Tris-HCl pH 6.8, 0.5% β-mercaptoethanol, 0.01% bromo-phenol blue). Large EVs released from about 10x106 microglia were

lysed directly in the same Laemmli buffer. Proteins were then separated by electrophoresis, blotted on nitrocellulose membrane filters and probed using mouse anti-Flotillin (1:1000, BD Biosciences), rabbit anti-Annexin A2 (1:5000, Abcam), mouse anti-GS28 (1:1000; BD Biosciences), rabbit anti-TOM20 (1:500; Santa Cruz Biotechnology) and mouse anti-Aβ 6E10 (1:1000; Biolegend, #SIG-39300). Photographic development was by chemiluminescence (ECL, Euroclone, Pero, Italy, or FEMTO, Thermo Fisher Scientific) according to the manufacturer's instructions, using a ChemiDoc™ Imager (Bio-Rad, Hercules, CA, USA). Western blot bands were quantified by ImageJ software. Aβ enrichment in Aβ-EVs compared to donor cells was calculated by quantifying 6E10 positive bands and normalizing on Flotillin

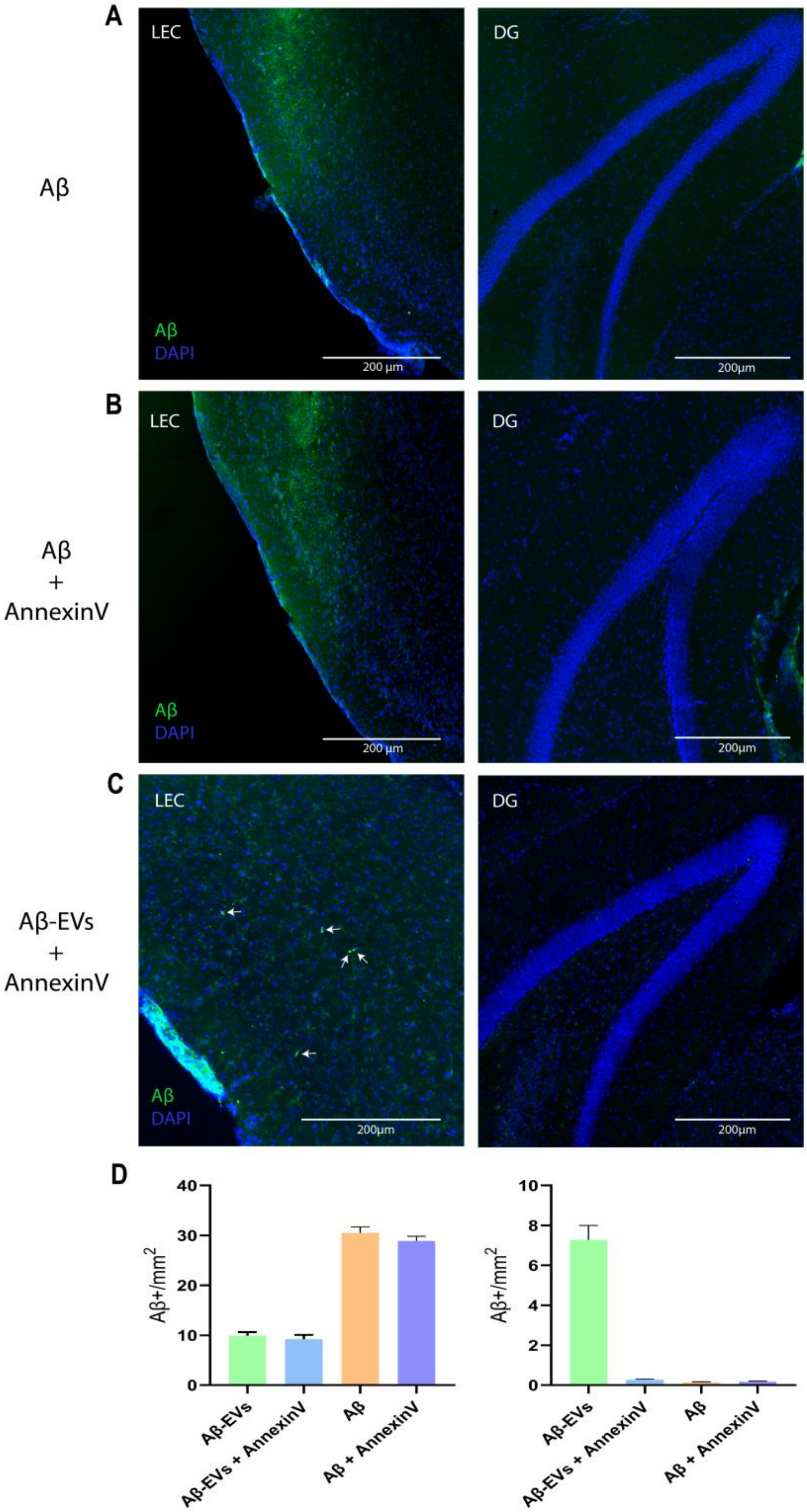

Supplemental figure 2. B) Aβ staining (in green) and DAPI staining (in blue) in the LEC at 1 h and DG at 24h following the injection, showing the localization of Aβ. (A,B, C left panels) Aβ staining (in green) and DAPI staining (in blue) 1h after Aβ (100nM), Aβ + Annexin V and Aβ EVs + Annexin V injection in the LEC shows the presence of Aβ in thee LEC. (A, B,C right panels) No traces of Aβ are detected 24 h after Aβ, Aβ + Annexin V and Aβ EVs + Annexin V injection. (D) Aβ staining quantification in LEC at 1 h (left panel) and in DG (right panel) at 24h after the injection. (bars represents the Aβ mean fluorescence intensity, n= 3 mice per group , error bars = SEM; $*p < 0.05$, one-way ANOVA.

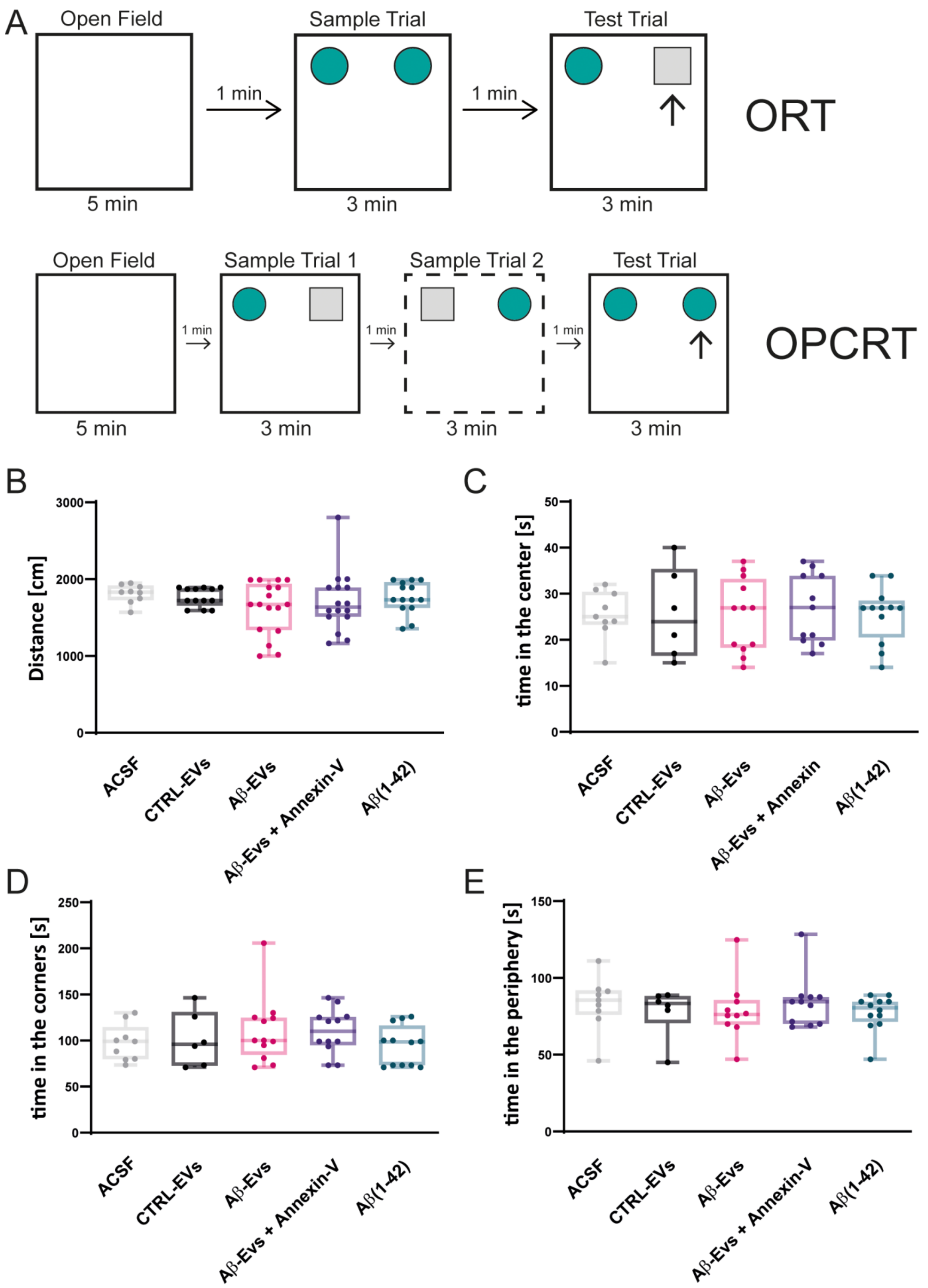
A
Open Field
1 min
Sample Trial
1 min
Test Trial
5 min
3 min
3 min
ORT
Open Field
Sample Trial 1
Sample Trial 2
Test Trial
1 min
1 min
1 min
5 min
3 min
3 min
3 min
OPCRT
B
Distance [cm]
ACSF
CTRL-EVs
Aβ-EVs
Aβ-Evs + Annexin-V
Aβ(1-42)
C
time in the center [s]
ACSF
CTRL-EVs
Aβ-Evs
Aβ-Evs + Annexin
Aβ(1-42)
D
time in the corners [s]
ACSF
CTRL-EVs
Aβ-Evs
Aβ-Evs + Annexin-V
Aβ(1-42)
E
time in the periphery [s]
ACSF
CTRL-EVs
Aβ-Evs
Aβ-Evs + Annexin-V
Aβ(1-42)

Supplementary figure 3. The novel object-place-context recognition test (OPCRT) and the novel object recognition test (ORT). (A) Schematic figure representing the novel object-place-context recognition test (OPCRT) on top and the novel object recognition test (ORT) below. The OPCRT is an EC-dependent task, used to assess the capability of the rodents to discriminate the novel object in relation to both its position and the surrounding context. This task requires the sequential presentation of objects in two different contexts. Mice were initially exposed to context 1 for 5 minutes (without objects), then they were free to explore for 3 minutes two different objects at two different locations (sample trial 1). Then, mice were put in context 2 for 3 minute to explore the same objects as before, at the opposite position with respect to sample trial 1 (sample trial 2). In the test trial, mice explored for 3 minutes again in the context 1 two copies of one of the two previously presented objects (familiar OPC association), one at the same location as sample trial 1, and the other in a novel location (novel OPC association). Therefore, as compared to the other tasks, the OPCRT requires the codification of both objects, their positions, and their contexts. The ORT is an hippocampal-dependent task. It is a classical behavioural test to evaluate learning and memory in mice, based on the innate curiosity of mice toward the novel object. The test relies on three sessions: one habituation session, one training session, and one test session. After 5 minutes of exploration in the "white" context, mice underwent a sample trial in which they were allowed to explore two copies of the same object for 3 minutes. Mice were then removed from the box and placed in a holding cage for 1 minute inter trial interval (i.t.i.) while the box was cleaned and configured for the following trial. In test trial mice were exposed to the same object as before, the familiar object (FO), and to a novel object (NO) and left free to explore the objects for 3 minutes. Because of the preference for novelty, once mice recognized the familiar object, it will tend to spend more time on the novel object. In all the above experiments, the exploration time was counted only when the mouse's nose was directed toward the object. Moreover, exploration was not considered when the mouse was immediately beside or even on top of the objects. To check for reliability, videos were re-scored by separate observer in a blind fashion and these scores were found to be consistent within 10% of the observers. For each task we converted observation scores into discrimination indices (discrimination index (D.I.)= (time at novel - time at familiar)/(time at novel + time at familiar)) to determine the rates that mice explored novel versus familiar objects/places/associations. Spontaneous locomotor activity was evaluated placing the mice in the center of an open-field arena (60x60x40cm), allowing mice to freely explore it for 5 minutes. The measured metrics were total distance traveled (in cm), time spent in the center and time spent in the corner of the arena. Tests were recorded using an IR camera and acquired videos were subsequently analyzed using the EthoVision XT 9 tracking software**. (B)** Average distance travelled by each mouse during the open field test. Kruskal Wallis test, H = 1.97, p = 0.58. **(C)** Average time spent in the center of the arena by each mouse during the open field test. Kruskal Wallis test, H = 0.69, p = 0.87. **(D)** Average time spent in the corners of the arena by each mouse during the open field. Kruskal Wallis test, H = 2.37, p = 0.50. (E) Average time spent in the periphery by each mouse in the open field test. Kruskal Wallis test, H = 0.90, p = 0.82.
